# Exploring localized ENZ resonances and their role in superscattering, wideband invisibility, and tunable scattering

**DOI:** 10.1038/s41598-024-51503-y

**Published:** 2024-01-18

**Authors:** Andriy E. Serebryannikov, Ekmel Ozbay

**Affiliations:** 1 Division of Physics of Nanostructures, ISQI, Faculty of Physics, Adam Mickiewicz University, 61-614 Poznan, Poland; 2 Nanotechnology Research Center (NANOTAM), Bilkent University, 06800 Ankara, Turkey; 3 National Institute of Materials Sciences and Nanotechnology (UNAM), Department of Physics, and Department of Electrical Engineering, Bilkent University, 06800 Ankara, Turkey

**Keywords:** Optics and photonics, Physics

## Abstract

While the role and manifestations of the localized surface plasmon resonances (LSPRs) in anomalous scattering, like superscattering and invisibility, are quite well explored, the existence, appearance, and possible contribution of localized epsilon-near-zero (ENZ) resonances still invoke careful exploration. In this paper, that is done along with a comparison of the resonances of two types in the case of thin-wall cylinders made of lossy and loss-compensated dispersive materials. It is shown that the localized ENZ resonances exist and appear very close to the zero-permittivity regime, i.e., at near-zero but yet negative permittivity that is similar to the ENZ modes in thin planar films. Near- and far-field characteristics of the superscattering modes are investigated. The results indicate that the scattering regimes arising due to LSPRs and localized ENZ resonances are distinguishable in terms of the basic field features inside and around the scatterer and differ in their contribution to the resulting scattering mechanism, e.g., in terms of the occupied frequency and permittivity ranges as well as the sensitivity to the wall thickness variations. When the losses are either weak or tend to zero due to the doping with gain enabling impurities, the sharp peaks of the scattering cross-section that are yielded by the resonances can be said to be embedded into the otherwise wide invisibility range. In the case of lossy material, a wide and continuous invisibility range is shown to appear not only due to a small total volume of the scatterer in the nonresonant regime, but also because high-Q superscattering modes are suppressed by the losses. For numerical demonstration, indium antimonide, a natural lossy material, and a hypothetical, properly doped material with the same real part of the permittivity but lower or zero losses are considered. In the latter case, variations of permittivity with a control parameter can be adjusted in such a way that transitions from one superscattering mode to another can be achieved. In turn, transition from the strong-scattering to the invisibility regime is possible even for the original lossy material. The basic properties of the studied superscattering modes may be replicable in artificial structures comprising natural low-loss materials.

## Introduction

Anomalous scattering has attracted a lot of attention in recent decades. It assumes that the strength of scattering can be untypically strong or untypically weak for a given size and material of the scatterer. The latter physical situation is often associated with *invisibility* and *cloaking*. There are two big classes of the approaches to invisibility. For the first of them, known as Transformation Optics, penetration of the incident electromagnetic wave into the region occupied by the scatterer is prevented by rerouting “rays” around it, by using a designed cloak which represents a finite-extent gradient metamaterial^1–4^. For the second of them, the invisibility of the scatterer for a far-zone observer is achieved while the field inside the dielectric scatterer can be rather strong, as happens in case of scattering cancellation^5–8^. Moreover, thin covers can totally change the overall scattering properties and yield invisibility, like in the case of carpet and mantle cloaks^8–11^. Next, the invisibility mechanisms based on plasmonic resonance^12^, single-mode interferences^13^, and specific interferences yielded by different modal/resonant regimes^14^ should be mentioned. *Superscattering*, a counterpart of the invisibility has been studied in various core-shell and multilayer structures^15–27^. It is often attributed to the effects exerted by (spoof) localized surface plasmon resonances (LSPRs)^28–31^. Invisibility and superscattering can be achieved in different frequency ranges in the same structure. Furthermore, switching between weak and conventional/strong scattering has been demonstrated at a fixed frequency, assuming that the scatterer comprises component(s) made of an actively tunable material^14,24,32–37^. In a wide sense, superscattering can be understood as scattering that is unexpectedly strong for the given size and material of the scatterer. From this point of view, Helmholtz-like electromagnetic resonance in the cavity-backed antennas^38,39^ can yield strong scattering at a small electrical size that formally allows for assigning it to the family of superscattering regimes. At the same time, epsilon-near-zero (ENZ) and near-zero-index physics and related applications have been extensively investigated^40–48^. In particular, materials and structures with the near-zero properties may enable a myriad of applications in different parts of electromagnetic spectrum, like tailoring the phase patterns^49^, control of nonlinearity of the particles in the proximity of an ENZ spherical scatterer^50^, substrates or host slabs to shape radiation of (nano-)antennas placed atop^51^ or inside it^52^ and for the scatterers placed above them^53^, and waveguide discontinuities enabling the dramatic changes in transmission^54^, to name a few. Among the physical effects that are directly related to the present work, the ENZ modes arising in ultrathin planar films should be mentioned^55–57^. Phase-change materials^58,59^, transparent conducting oxides^60,61^ and polar dielectrics^62–65^ are the candidates to be used in ENZ components.

In the present paper, the existence of the localized ENZ resonances is confirmed and their contribution to anomalous scattering on thin-wall cylinders made of a Drude dispersive model is explored by using a comparison with LSPRs. The frequency range is set within the THz range, and the geometrical parameters of the scatterers are chosen so that their electrical sizes are changed from deeply subwavelength to those of the order of free-space wavelength. It will be shown that a wide and continuous invisibility range may appear when the high-Q superscattering modes arising due to LSPRs or localized ENZ resonances are suppressed by the losses. In turn, when the losses are weak or zero, such resonances may lead to sharp maxima of the scattering cross-section. We assume here that the effect of losses can be partially or fully compensated by gain impurities, or can be negligible in case of the properly designed metallo-dielectric composites. It will be shown that the superscattering modes of two types are distinguishable. In particular, for the first of them which are connected with a LSPR, azimuthal electric field inside the cylinder can be dramatically enhanced, as is typical for resonances of this type^28–30^. The second of them represent the localized ENZ resonances, whose connection to the ENZ modes in planar structures^56^ is, generally speaking, *similar* to the connection of LSPRs in cylindrical/wrapped structures to surface plasmons in planar structures. In fact, the term *localized ENZ resonances* highlights the analogy between them and the aforementioned ENZ modes, on the one hand, and another pair of phenomena, i.e., LSPRs and Surface Plasmons, on the other hand. Indeed, it is demonstrated here that the localized ENZ resonances appear in the near-zero negative permittivity regime, showing rather strong radial electric and axial magnetic fields inside the cylinder; this makes their connection to ENZ modes evident, for which a transverse electric field inside a thin planar film is strongly enhanced^56^. Therefore, thin-wall cylinders are needed to obtain the localized ENZ resonances. Moreover, we will show that scattering can be on-off switched, or switched between two superscattering modes or between strong- and weak-scattering regimes, if the thin-wall cylinder is made of an actively tunable material. Notably, results related to thin-wall structures made of metals were reviewed in Ref.^66^.

## Scattering scenarios enabled by localized ENZ and surface plasmon resonances

**Figure 1:**
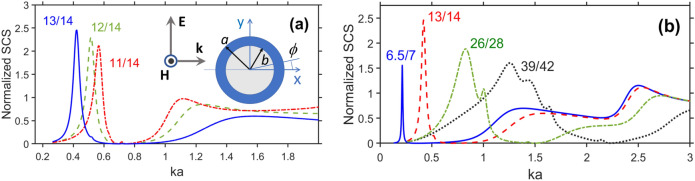
Normalized total scattering cross section, $\sigma _t$, for three thin-wall cylinders at $\varepsilon _c=\varepsilon _s$ for (**a**) $b=11~\upmu \text{ m }$ (dash-dotted red line), $12~\upmu \text{ m }$ (dashed green line), and $13~\upmu \text{ m }$ (solid blue line), $a=14~\upmu \text{ m }$; (**b**) $b=6.5~\upmu \text{ m }$ and $a=7~\upmu \text{ m }$ (solid blue line), $b=13~\upmu \text{ m }$ and $a=14~\upmu \text{ m }$ (dashed red line), $b=26~\upmu \text{ m }$ and $a=28~\upmu \text{ m }$ (dash-dotted green line), and $b=39~\upmu \text{ m }$ and $a=42~\upmu \text{ m }$ (dotted black line); geometry is shown in the inset in plot (**a**). *b*/*a* is shown near the curves.

In this section, we overview the basic scattering features of thin-wall hollow cylinders made of a Drude-dispersive lossy material. General geometry is shown in the inset in Fig. 1a. The *p*-polarized plane wave is incident from the side of negative *x* values, i.e., $\phi =\pi$ corresponds to the incidence direction. We restrict consideration to *p*-polarization, because the effects which we are interesting in are observed only in this case. For the sake of definiteness, we first consider InSb, a phase-change material as the cylinder material which has recently been used in various structures^33,67–70^. In the range of transition from the metallic phase to the insulator phase, its permittivity is described by the Drude model^68,71^, as follows:

1 $$\begin{aligned} \varepsilon _s=\varepsilon _{\infty }-\omega _p^2/(\omega ^2-i\gamma \omega ), \end{aligned}$$

where high-frequency permittivity $\varepsilon _{\infty }=15.68$, the damping constant $\gamma =\pi \times {10^{11}}~\text{ rad } \text{ s}^{-1}$, plasma frequency

$\omega _p=\sqrt{N{q_e}^2/0.015\varepsilon _0{m_e}}$, charge and mass of electron are $q_e=-1.6\times {10^{-19}}$ C and $m_e=9.11\times {10^{-31}}$ kg, respectively, the intrinsic carrier density (in $\text{ m}^{-3}$) $N=5.76\times {10^{20}}T^{3/2}\text{ exp }(-E_g/{2k_{B}T})$, the band-gap energy $E_g=0.26$ eV, Boltzman constant is $k_B=8.62\times {10^{-5}}~\text{ eV } \text{ K}^{-1}$, *T* is temperature, and $\varepsilon _0=8.854\times 10^{-12}~\text{ F/m }$. Further, we take $T=295~\text{ K }$ in Eq. (1), so $\text{ Re }\varepsilon _s=0$ at $f=2.45355$ THz. The exception is related to section “Transitions from variations of the control parameter” where *T* is assumed to be variable.

Figure 1a presents $\sigma _t$ vs. *ka* (*k* is free-space wavenumber) for $a=14~\upmu \text{ m }$ when the permittivity of the cylinder material $\varepsilon _c$ is equal to $\varepsilon _s$ given by Eq. (1). Details of the calculation of $\sigma _t$ are given in Appendix. As expected, the basic features include (i) strong resonant scattering in the deeply subwavelength regime, i.e., at $ka<0.6$ and (ii) wide range of invisibility in the vicinity of $ka=0.8$ (e.g., $0.6<ka<1$ at $b=13~\upmu \text{ m})$. The former is connected with an LSPR; the latter occurs in the *ka* range where $\vert \text{ Re }\varepsilon _c\vert$ is relatively small, and so the existence of the weak-scattering regime beyond the resonances looks quite natural. However, as explained below, the latter needs additional clarifying. The strong-scattering regime at the first maximum of $\sigma _t$ is spectrally downshifted with increase of *b*, so it appears at $ka=0.565$, 0.515, 0.42 ($\text{ Re }\varepsilon _c=-9.75$, $-14.9$, and $-30$), for $b/a=11/14$, 12/14, and 13/14, respectively. In turn, the weak scattering regime, say, with $\sigma _t<10^{-2}$ is achieved at $0.59<ka<0.92$ ($-7.6<\text{ Re }\varepsilon _c<6.1$) when $b/a=13/14$, $0.65<ka<0.83$, except for the vicinity of $ka=0.716$ (the vicinity of $\text{ Re }\varepsilon _c=-0.1$) when $b/a=12/14$, and at $0.671<ka<0.708$ and $0.725<ka<0.8$ when $b/a=11/14$. Clearly, the weak scattering results here not from scattering cancellation^5–8^, because the core is vacuum. Notably, in Fig. 1a $\text{ Re }\varepsilon _c=1$ at $ka=0.743$ and $\text{ Re }\varepsilon _c=0$ at $ka=0.719$. Similar results obtained for $a=28~\upmu \text{ m }$ and $42~\upmu \text{ m }$ can be found in Supplementary Information, see Fig. S1. Figure 1b presents $\sigma _t$ vs. *ka* for several pairs of (*b*, *a*) but now $a\neq\text{ const }$. From the comparison of Fig. 1a,b, we observe that the invisibility regime is conserved for a quite arbitrary choice of *b* and *a*, and can appear even for an electrically non-small scatterer if $\text{ Re }\varepsilon _c={1}$ (e.g., at $2ka\approx 4.5$ for $b/a=39/42$). As expected, the smaller and the thinner the cylinders are, the wider the range of weak scattering will be.

**Figure 2:**
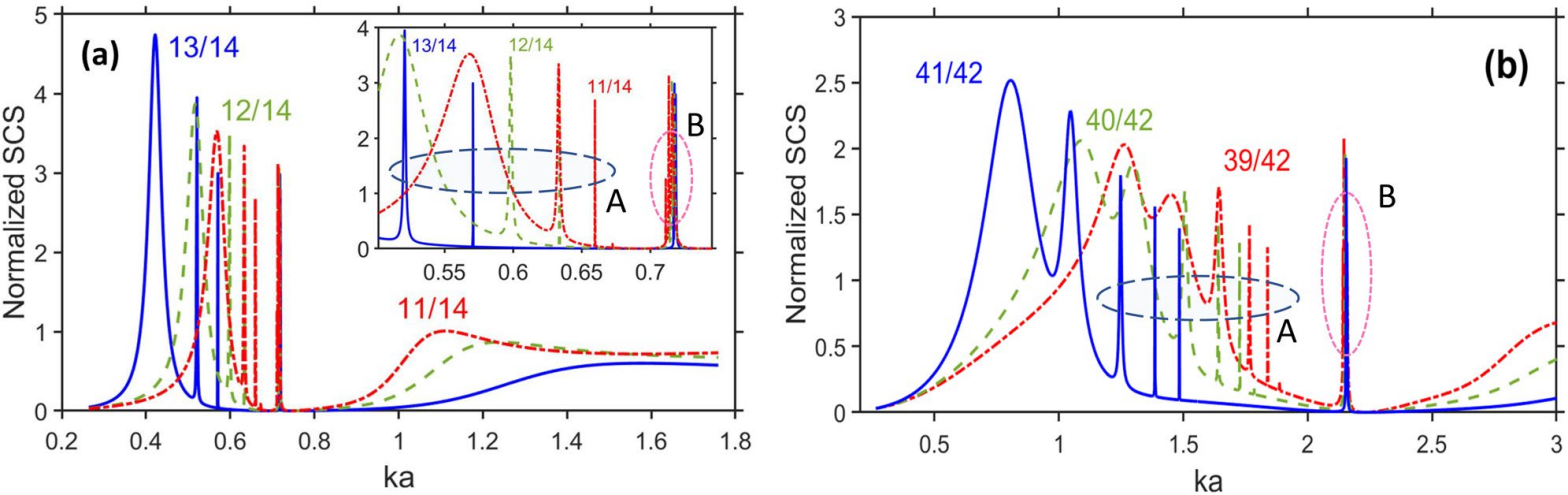
Normalized total scattering cross section, $\sigma _t$, for cylinders made of lossless material, $\varepsilon_c =\text{ Re }\varepsilon _s$, for (**a**) $b=11~\upmu \text{ m }$ (dash-dotted red line), $b=12~\upmu \text{ m }$ (dashed green line), and $b=13~\upmu \text{ m }$ (solid blue line), at $a=14~\upmu \text{ m }$; inset shows the fragment for the range $0.5<ka<0.75$; (**b**) same as (**a**) but for $b=39~\upmu \text{ m }$, $40~\upmu \text{ m }$, $41~\upmu \text{ m }$, at $a=42~\upmu \text{ m }$. The ratios of *b*/*a* are shown near the curves.

To further explore the basic scattering features, we investigate scattering on the cylinders made of an ideal lossless material with permittivity $\varepsilon _c=\text{ Re }\varepsilon _s$. For the sake of simplicity, we assume that it can be obtained by using either the gain impurities to compensate for the losses in a natural material or, alternatively, a metallo-dielectric composite, depending on the chosen frequency range. The results for $\sigma _t$ vs. *ka* are presented in Fig. 2a for the same values of *b* and *a* as in Fig. 1a, whereas in Fig. 2b we use the value of *a* that is same as in one of the cases in Fig. 1b. Similar results for another value of *a* taken from Fig. 1b are presented in Fig. S2 in Supplementary Information. The main difference of the results in Fig. 2 is that there are sharp peaks of $\sigma _t$ at $\varepsilon _c=\text{ Re }\varepsilon _s$ in the same *ka*-subranges, in which weak scattering occurs at $\varepsilon _c=\varepsilon _s$. These peaks appear due to the effects exerted by superscattering modes. Among them, the modes of the two types should be distinguished. The scattering modes denoted as the modes of the type A are observed in Fig. 2a in the *ka*-range that corresponds to $-14.2<\text{ Re }\varepsilon _c<-2.97$, and the scattering modes denoted as the modes of the type B are observed in the *ka*-range corresponding to $-0.4<\text{ Re }\varepsilon _c<0$. They are connected with LSPRs and localized ENZ resonances, respectively. This makes it evident that a rather large width and continuity of the invisibility range in Fig. 1 results, among others, from (most of) the superscattering modes being dark modes. For $\text{ Im }\varepsilon _c=0$, we can obtain several narrow invisibility ranges that are *intermittent* with the strong-scattering regimes. It can be said that, in some senses, superscattering modes are *embedded* into the invisibility range. The lowest-*f* maximum of $\sigma _t$ manifests itself similarly to the case of $\varepsilon _c=\varepsilon _s$. The second and third maximums of $\sigma _t$ are connected with the modes of the type A that appear at $ka=0.6332$ and 0.6596 ($\text{ Re }\varepsilon _c=-4.55$ and $-2.97$) when $b/a=11/14$, at $ka=0.598$ and $ka=0.6337$ ($\text{ Re }\varepsilon _c=-7$ and $-4.5$) when $b/a=12/14$, and at $ka=0.521$ and $ka=0.5707$ ($\text{ Re }\varepsilon _c=-14.2$ and $-9.23$) when $b/a=13/14$. These modes are moderately sensitive to the variations in *b* and *a*. In turn, the modes of the type B appear only near $\text{ Re }\varepsilon _c=0$. The corresponding sharp peaks of $\sigma _t$ indicate the presence of resonances coupled to the far field. The results for $a=42~\upmu \text{ m }$ are presented in Fig. 2b. It is seen that the main features here are the same as for $a=14~\upmu \text{ m }$, except for that the superscattering modes can increase within the range of significant scattering, but not necessarily within the invisibility range. Finally, the results for $a=28~\upmu \text{ m }$ can be found in Supplementary Information; see Fig. S2 therein.

**Figure 3:**
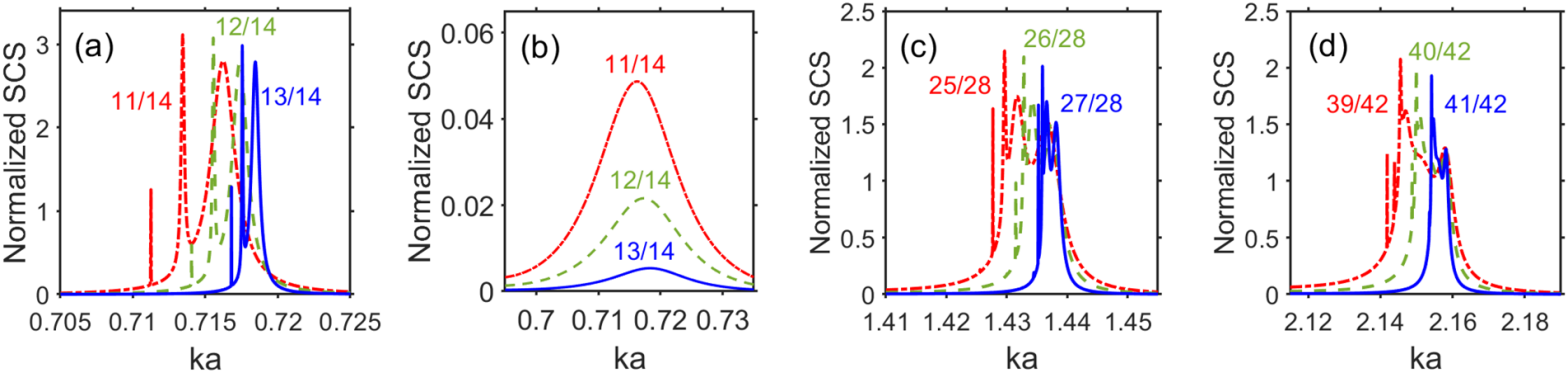
Normalized total scattering cross section, $\sigma _t$, for scattering modes of type B: (**a**) $\varepsilon _c=\text{ Re }\varepsilon _s$ when $b=11~\upmu \text{ m }$ (dash-dotted red line), $b=12~\upmu \text{ m }$ (dashed green line), $b=13~\upmu \text{ m }$ (solid blue line), at $a=14~\upmu \text{ m }$; (**b**) same as (**a**) but for $\varepsilon _c=\varepsilon _s$; (**c**) same as (**a**) but for $b=25~\upmu \text{ m }$, $26~\upmu \text{ m }$, and $27~\upmu \text{ m }$ at $a=28~\upmu \text{ m }$; (**d**) same as (**a**) but for $b=39~\upmu \text{ m }$, $40~\upmu \text{ m }$, and $41~\upmu \text{ m }$, at $a=42~\upmu \text{ m }$.

**Figure 4:**
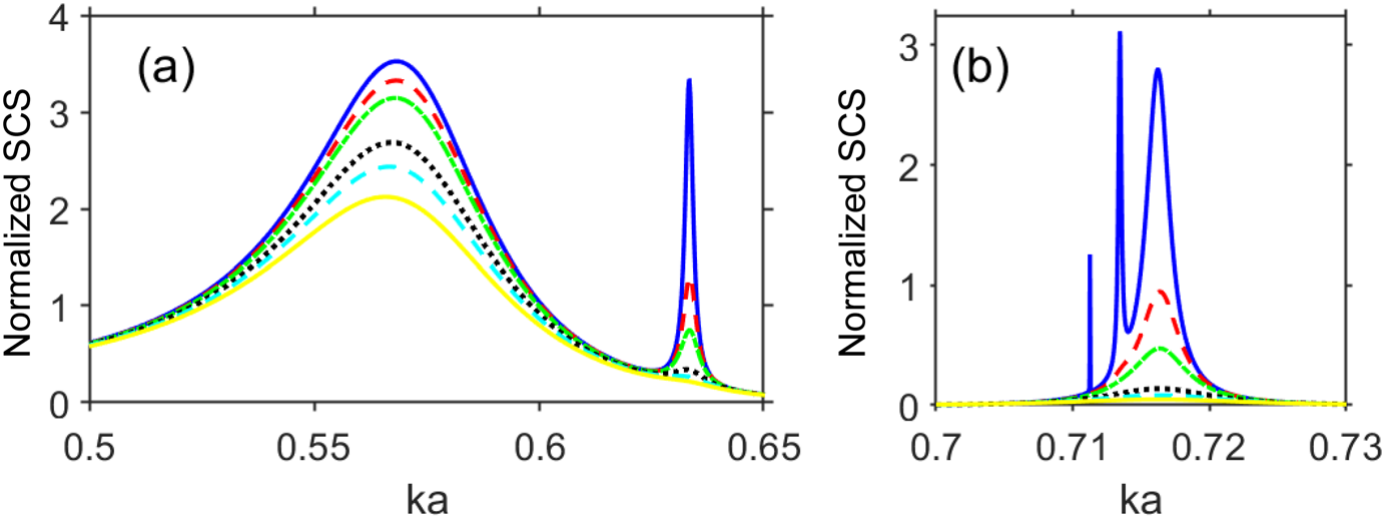
Normalized total scattering cross section, $\sigma _t$, for (**a**) lowest-frequency mode and one of the modes of type A, and (**b**) modes of type B, for various values of *C* in $\text{ Im }\varepsilon _c=C\text{ Im }\varepsilon _s$: $C=0$—solid blue line, $C=0.1$—dashed red line, $C=0.2$—dash-dotted green line, $C=0.5$—dotted black line, $C=0.7$—dashed cyan line, $C=1$—solid yellow line; $\text{ Re }\varepsilon _c=\text{ Re }\varepsilon _s$, $b=11~\upmu \text{ m }$ and $a=14~\upmu \text{ m }$; (**a**) the lowest-frequency mode and one of the high-Q modes of the type A, (**b**) modes of the type B. .

Figure 3 presents the details of $\sigma _t$ for the modes of the type B at different values of *b* and *a*. In Fig. 3a, we present the results obtained at $\varepsilon _c=\text{ Re }\varepsilon _s$ and $a=14~\upmu \text{ m }$. The peaks of $\sigma _t$ are observed, which are yielded by the localized ENZ resonances. For comparison, in Fig. 3b, the results are presented for the same values of *b* and *a* as in Fig. 3a but for $\varepsilon _c=\varepsilon _s$. As observed, there is no signature of superscattering modes in $\sigma _t$. In Fig. 3c, we take $a=28~\upmu \text{ m }$, so instead of $2a/\lambda \approx {0.23}$ as in Fig. 3a superscattering modes occur nearly at $2a/\lambda \approx {0.455}$. In spite of this difference, the basic features remain the same. Finally, in Fig. 3d, $2a/\lambda \approx {0.68}$ for $a=42~\upmu \text{ m }$. In addition, similarly to Fig. 3a,b, the sharp peaks of $\sigma _t$ dramatically disappear at $a=28~\upmu \text{ m }$ and $42~\upmu \text{ m }$, if $\varepsilon _c=\text{ Re }\varepsilon _s$ is replaced by $\varepsilon _c=\varepsilon _s$ (not shown). The peaks of the type B are located at negative but close-to-zero values of $\varepsilon _c$. In Fig. 3d, the closest maximum corresponds to $\varepsilon _c=-1.4\times {10^{-3}}$ ($ka=2.1582$), which is unprecedently close to $\text{ Re }\varepsilon _c=0$. In Fig. 3a,c, such a peak appears at $\text{ Re }\varepsilon _c=-0.1406$ ($ka=0.7162$) and $\text{ Re }\varepsilon _c=-4.28\times {10^{-2}}$ ($ka=0.7184$), respectively. Since the maximums of type B always appear at $\text{ Re }\varepsilon _c<0$, they can be said to be different from the effects studied in Refs.^49,50,72^ at $\text{ Re }\varepsilon _c\ge 0$. However, they may be similar in some senses to the ENZ modes, which appear in ultrathin planar ENZ slabs at $\text{ Re }\varepsilon _c<0$^56^, but the analysis of the field distribution is needed to clarify it.

Figure 4 presents $\sigma _t$ vs. *ka* at different values of $\text{ Im }\varepsilon _c$. As observed, the different scattering modes show different sensitivity to the same variations of $\text{ Im }\varepsilon _c$. Expectedly, the narrower (i.e., higher-*Q*) maxima of $\sigma _t$ are stronger sensitive to the variations in $\text{ Im }\varepsilon _c$. The lowest-frequency resonance still well manifests itself also when $\text{ Im }\varepsilon _c=\text{ Im }\varepsilon _s$, whereas the superscattering modes of the types A and B either are dramatically weakened or totally disappear. Therefore, these modes can be either bright or dark, depending on the imaginary part of permittivity; also see Fig. S3 in Supplementary Information. It is noteworthy that the superscattering modes can be selectively amplified when $\text{ Im }\varepsilon _c>0$ (not shown), see Fig. S4 in Supplementary Information. Notably, the modes of the type B are approximately *scalable* by varying *a*, because their spectral locations are predetermined by the material properties (i.e., the vicinity of $\text{ Re }\varepsilon _c=0$ is required). In turn, for the modes of the type A, the effect of variations in *a* on their number and spectral locations is more complicated.

## Far- and near-field properties of superscattering modes

**Figure 5:**
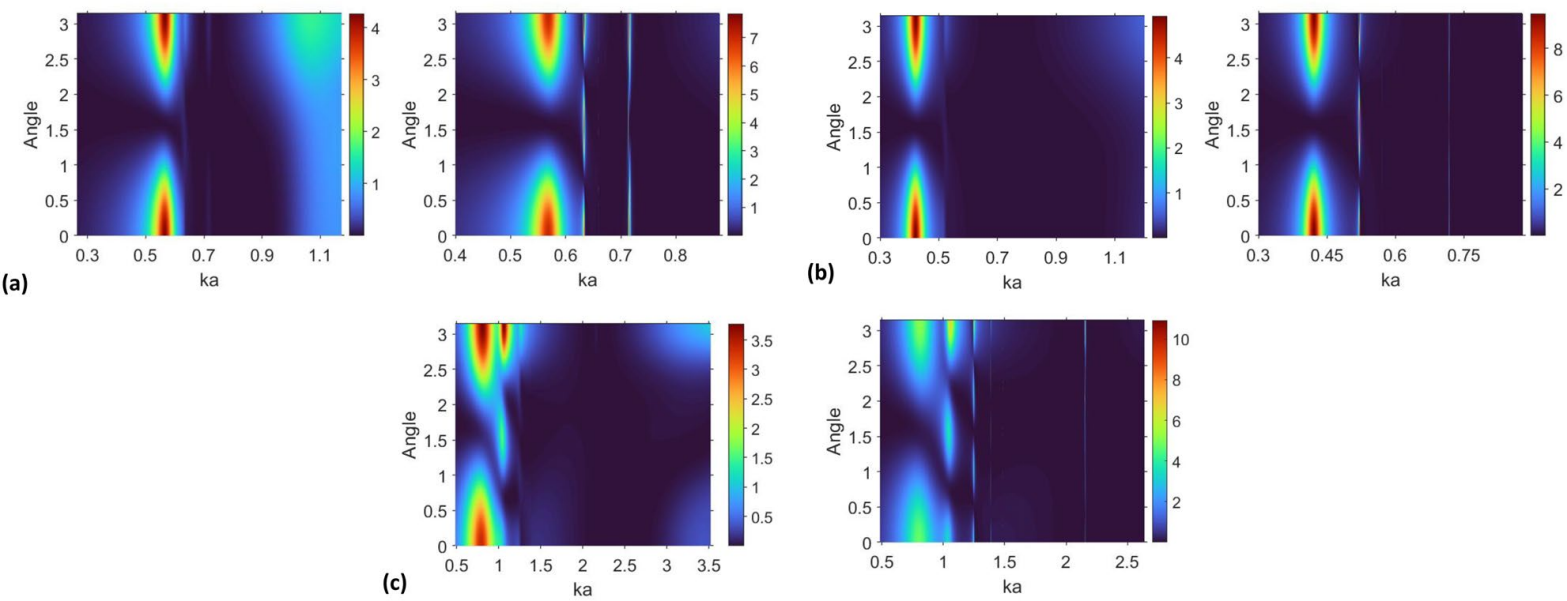
Normalized $\phi$-dependent scattering cross section (a.u.), $\sigma (\phi )$, for (**a**) $b=11~\upmu \text{ m }$ and $a=14~\upmu \text{ m }$, at $\varepsilon _c=\varepsilon _s$ (left plot) and $\varepsilon _c=\text{ Re }\varepsilon _s$ (right plot); (**b**) $b=13~\upmu \text{ m }$ and $a=14~\upmu \text{ m }$, $\varepsilon _c=\varepsilon _s$ (left plot) and $\varepsilon _c=\text{ Re }\varepsilon _s$ (right plot); (**c**) $b=41~\upmu \text{ m }$ and $a=42~\upmu \text{ m }$; $\varepsilon _c=\varepsilon _s$ (left plot) and $\varepsilon _c=\text{ Re }\varepsilon _s$ (right plot); the angle $\phi$ (ordinate axis) is measured from the *x*-axis in the counter-clockwise direction (see inset in Fig. 1a) and shown here in units of $\pi$.

In this section, we investigate a $\phi$-dependent scattering cross-section, $\sigma (\phi )$, which does not accumulate the effects of all observation angles. Figure 5 presents the results on the $(ka,\phi )$-plane. The angular behavior corresponding to the lowest-*f* maximum (in all plots) indicates the dominant contribution of the dipolar space harmonics [$\vert {l}\vert =1$ in the field expansions, see Eqs. (2)–(4)]. As observed, the difference between the cases of $\varepsilon _c=\varepsilon _s$ and $\varepsilon _c=\text{ Re }\varepsilon _s$ for this mode occurs only in that $\sigma (\phi )$ is smaller for the former. The results presented for $\varepsilon _c=\varepsilon _s$ indicate that there are just weak signatures of the superscattering modes of the types A and B, whose effects on $\sigma (\phi )$ are seen for $\varepsilon _c=\varepsilon _s$ at $ka>0.62$ for $b=11~\upmu \text{ m }$ and $a=14~\upmu \text{ m }$, at $ka>0.5$ for $b=13~\upmu \text{ m }$ and $a=14~\upmu \text{ m }$, and at $ka>1.2$ for $b=41~\upmu \text{ m }$ and $a=42~\upmu \text{ m }$. There are different numbers of the maxima over $\phi$ for different scattering modes and, therefore, for different dominant $\vert {l}\vert$. As mentioned above, suppression of most of these modes is crucial for the wideness and continuity of the invisibility range when $\varepsilon _c=\varepsilon _s$. Note that some scattering features arising due to the closely spaced and/or narrow resonance peaks can be poorly distinguishable in Fig. 5, because of being too narrow as compared to the width of the considered *ka*-range.

**Figure 6:**
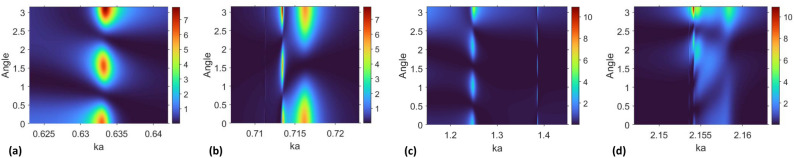
Details of $\sigma (\phi )$ from Fig. 5a at $\varepsilon _c=\text{ Re }\varepsilon _s$, in the vicinity of (**a**) $ka=0.63$ and (**b**) $ka=0.715$ when $b/a=11~\upmu \text{ m }$ and $a=14~\upmu \text{ m }$, and in the vicinity of (**c**) $ka=1.3$ and (**d**) $ka=2.155$ when $b=41~\upmu \text{ m }$ and $a=42~\upmu \text{ m }$; angle $\phi$ is measured from the *x*-axis in the counter-clockwise direction and shown here in units of $\pi$. (**a**,**c**) scattering modes A connected with LSPRs; (**b**,**d**) scattering modes B connected with localized ENZ resonances.

The magnified fragments of Fig. 5a,c are presented in Fig. 6. You can see that $\vert {l}\vert =2$ dominates in the far field at $ka=0.633$ ($\text{ Re }\varepsilon _c=-4.5596$) in Fig. 6a, whereas $\vert {l}\vert =2$ and $\vert {l}\vert =1$ at $ka=0.7135$ ($\text{ Re }\varepsilon _c=-0.2614$) and $ka=0.7162$ ($\text{ Re }\varepsilon _c=-0.1428$) do so in Fig. 6b. In Fig. 6c, multipolar components with $\vert {l}\vert =3$ and $\vert {l}\vert =4$ are dominant, respectively, at $ka=1.248$ ($\text{ Re }\varepsilon _c=-31.18$) and $ka=1.3859$ ($\text{ Re }\varepsilon _c=-22.3$). In the case shown in Fig. 6d, the backward scattering prevails and $\sigma (\phi )$ results mainly from the superposition of space harmonics with different values of $\vert {l}\vert$.

**Figure 7:**
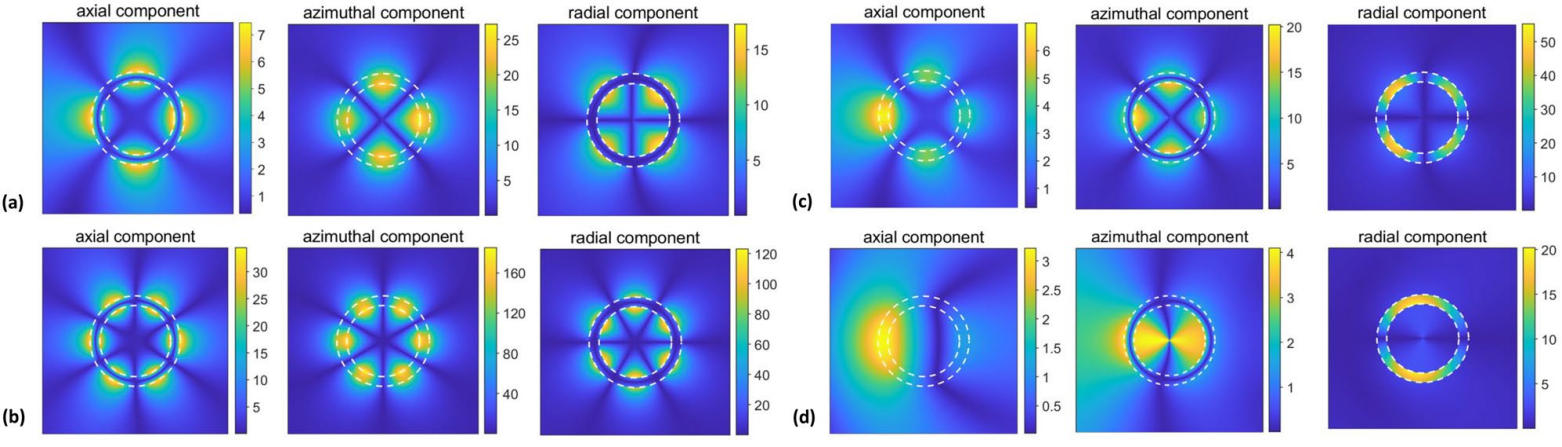
$\vert {H_z}\vert$ (left), $\vert {E_{\phi }}\vert$ (middle), and $\vert {E_r}\vert$ (right) for $b=11~\upmu \text{ m }$, $a=14~\upmu \text{ m }$ and $\varepsilon _c=\text{ Re }\varepsilon _s$ at (**a**) $ka=0.6332$, (**b**) $ka=0.6596$, (**c**) $ka=0.7135$, and (**d**) $ka=0.7162$. (**a**) and (**b**) correspond to the scattering modes of the type A, (**c**) and (**d**) correspond to the scattering modes of the type B. Cylinder walls are shown by two concentric dashed white lines.

Next, let us consider near-field behavior for the selected spectral regimes, assuming unitary magnitude of the incident wave’s magnetic component. Figure 7 presents the field distributions within and around the cylinder for the strong-scattering regimes associated with the modes of both type A [plots (a,b)] and type B [plots (c,d)]. The values of *ka* are chosen to correspond to the maxima of $\sigma _t$ and $\sigma (\phi )$ obtained for $b=11~\upmu \text{ m }$ and $a=14~\upmu \text{ m }$. As observed, strong enhancement occurs for the field components either outside the cylinder’s wall or inside it. The modes of type A and type B are easily recognizable, because of having principally different field features. In particular, the modes in the vicinity of $ka=0.64$ and the ones in the vicinity of $ka=0.715$ show very different field distributions, despite similar manifestations in the far field. In Fig. 7a,b, $\vert {E_{\phi }}\vert$ inside the wall is substantially stronger than for the incident wave, whereas in Fig. 7c,d such enhancement occurs rather for $\vert {H_z}\vert$ and $\vert {E_r}\vert$. This difference is predetermined by the basic properties of LSPRs and localized ENZ resonances, which should be distinguished from each other. Therefore, the differences between the superscattering modes of types A and B occur and are not restricted to their spectral location and sensitivity to the variations in *a* and *b*. It is noteworthy that the strong enhancement of $\vert {E_r}\vert$ for the modes of type B, which correspond to localized ENZ resonances, is an analog of the strong enhancement of the transverse electric field in case of ENZ modes in ultrathin films, e.g., see behavior of $\vert {E_y}\vert$ in^56^. Therefore, the aforementioned ultrathin planar films can be said to be a planar version of our thin-wall cylindrical structures and vice versa. Note that in the presented examples, the dominant contribution of the harmonics with $\vert {l}\vert =2$ in Fig. 7a and $\vert {l}\vert =3$ in Fig. 7b for the modes of type A. In turn, for the modes of type B, we observe the dominant contribution of $\vert {l}\vert =2$ in Fig. 7c and $\vert {l}\vert =1$ in Fig. 7d, so that the two spectrally close maxima of $\sigma _t$ correspond to the modes having principally different field distributions. It is remarkable that a smaller $\vert {l}\vert$ corresponds to the peaks located closer to the frequency value, at which $\text{ Re }\varepsilon _c=0$ (as exemplified by comparison of Fig. 7c,d), so that in the limiting case of $\text{ Re }\varepsilon _c=0$ we might expect obtaining the azimuthally uniform phase distribution in the cylinder. It is in the coincidence with the general theory of ENZ media, which highlights the zero phase advancement at $\varepsilon _c=0$. Finally, it should be noted that strong electric field can be obtained in ENZ shells also at $\text{ Re }\varepsilon _c>0$, like it occurs in the evanescent-mode regime in Ref.^50^, but it may happen that there would be no coupling to the far field, in contrast to the studied modes of type B.

**Figure 8:**
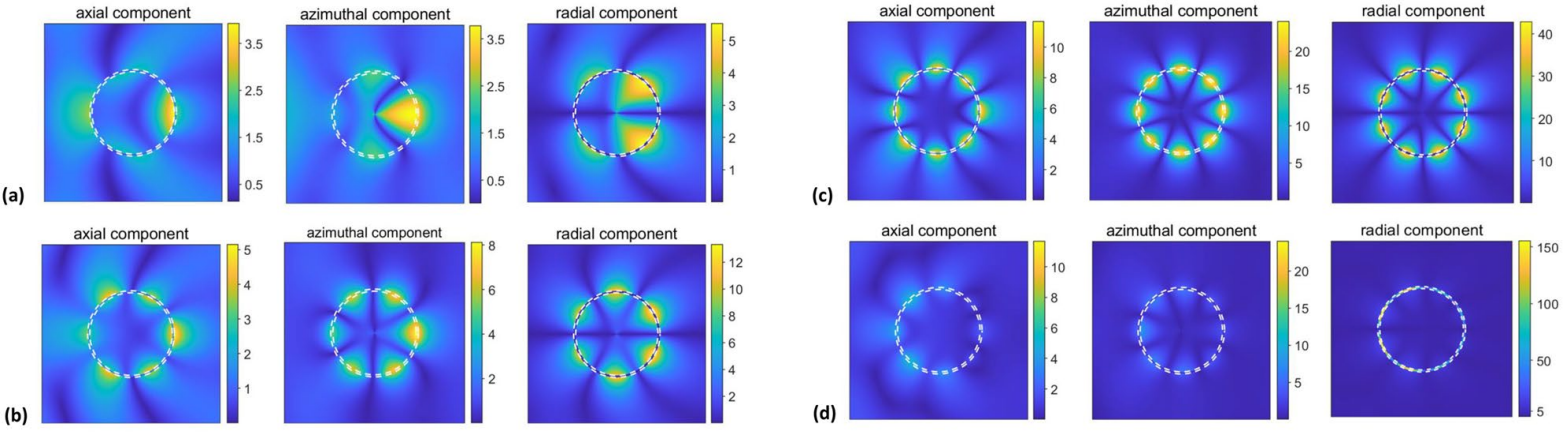
$\vert {H_z}\vert$ (left), $\vert {E_{\phi }}\vert$ (middle), and $\vert {E_r}\vert$ (right) for $b=40~\upmu \text{ m }$ and $a=42~\upmu \text{ m }$ and $\varepsilon _c=\text{ Re }\varepsilon _s$ at (**a**) $ka=1.3104$, (**b**) $ka=1.5065$, (**c**) $ka=1.6402$, and (**d**) $ka=2.1501$. (**a**) corresponds to the second-lowest (i.e., still not narrow) scattering maximum, (**b**) and (**c**) do so for the high-Q modes of the type A, and (**d**) for the modes of the type B. Cylinder walls are shown by two concentric, closely spaced, dashed white lines.

Now, let us take a larger value of *a*, i.e., $a=42~\upmu \text{ m }$, like in Figs. 2b and 3d, in order to obtain the regime with $2a\propto \lambda$ at $\text{ Re }\varepsilon _s\approx {0}$. A bit smaller value of *b* than in Fig. 5c, $b=40~\upmu \text{ m }$, is taken for the better visibility of the basic features. Four examples are presented in Fig. 8. In Fig. 8a, the field distributions are shown for the second-lowest (LSPR related) maximum arising at $ka\approx 1.31$. Contribution of the space harmonics with small but different $\vert {l}\vert$ leads here to some asymmetry between forward and backward scattering. In Fig. 8b,c, the field distributions are presented for the modes of the type A that appear in the vicinity of $ka=1.5$ and $ka=1.64$, where the space harmonics $\vert {l}\vert =3$ and $\vert {l}\vert =4$ are dominant, respectively. Finally, the results in Fig. 8d are presented for one of the modes of the type B, which appears in the vicinity of $ka=2.15$. In this case, the space harmonics with $\vert {l}\vert =4$ are the main contributors, although the effect of other harmonics cannot be said to be negligible. The values of $\vert {l}\vert$ retrieved from the results in Figs. 5 and 6 are in agreement with the results in Fig. 7. In some senses, the near-field features are *imaged* in the far field. In other words, for the near-fields of LSPRs and localized ENZ resonances, each maximum within/near the cylinder unambiguously corresponds to one of the maxima of $\sigma (\phi )$. In particular, this follows from the comparison of Fig. 6a with Fig. 7a, Fig. 6b with Fig. 7c,d, and other similar (but not shown) results. Since the modes of the type A are connected with LSPRs, it is not surprising that the features observed in Figs. 7a,b and 8a–c can be similar to those observed in various cylindrical structures supporting LSPRs^28–31^. Some similarities with Whispering-Gallery Modes^73–78^ should also be highlighted.

## Transitions from variations of the control parameter

**Figure 9:**
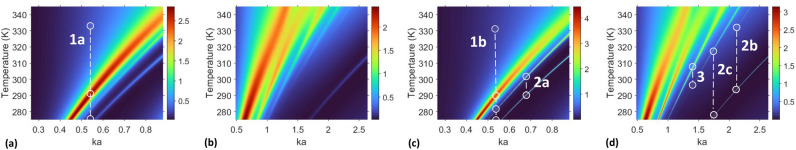
Normalized total scattering cross section, $\sigma _t$, in (*ka*,*T*)-plane for (**a**) $b=11~\upmu \text{ m }$ and $a=14~\upmu \text{ m }$, (**b**) $b=41~\upmu \text{ m }$ and $a=42~\upmu \text{ m }$, when $\varepsilon _c=\varepsilon _s$; (**c**) $b=11~\upmu \text{ m }$ and $a=14~\upmu \text{ m }$, (**d**) $b=41~\upmu \text{ m }$ and $a=42~\upmu \text{ m }$, when $\varepsilon _c=\text{ Re }\varepsilon _s$. 1a, 1b, 2a, 2b, 2c, 3 indicate different transitions from one scattering mode to another.

In this section, we study the behavior of $\sigma _t$ vs. *ka* under continuous variations of *T* that affect the carrier density, see Eq. (1). We assume that the materials with $\varepsilon _c=\text{ Re }\varepsilon _s$ may keep the similar sensitivity to the variations in *T*, as their undoped lossy counterparts have $\varepsilon _c=\varepsilon _s$. However, even if the sensitivity of the resulting impurity-containing material to the *T* variations will differ from that of the original lossy material, it is expected to be capable of diverse transitions from one scattering mode to another. Figure 9 presents the simulation results for $\sigma _t$ plotted on the (*ka*,*T*)-plane, for both cases of $\varepsilon _c=\varepsilon _s$ and $\varepsilon _c=\text{ Re }\varepsilon _s$. Note that some details may be not distinguishable, because the entire *ka* range hosting the studied modes is much wider than the *ka* range hosting, for example, the modes of the type B. At $\varepsilon _c=\varepsilon _s$, $\text{ max }\sigma _t$ which correspond to the lowest-*f* LSPR mode is conserved and spectrally upshifted while increasing *T*. Obtaining $\Delta {ka}=0.4$ for its spectral location needs an increase of *T* about $55~\text{ K }$. It leads to the spectral shift of invisibility range(s). The observed behavior is predetermined by the manner in which $\varepsilon _s$ depends on *T*, according to Eq. (1). By varying *T*, the transition from invisibility to strong scattering and then back to weak scattering can be easily achieved even at $\varepsilon _c=\varepsilon _s$. For instance, in Fig. 9a, one such transition is indicated by 1a. In fact, there is a lot of leeway in the choice of a *ka* value, whereas variation $\Delta {T}$ from $15~\text{ K }$ to $30~\text{ K }$ can be sufficient for each step in this two-step transition. The increase of *a* leads to there possibly being more modes, which can be involved to the transition scenarios, compare Fig. 9b with Fig. 9a. However, the fact that a rather strong scattering occurs in Fig. 9b within a wider *ka* range needs a careful adjustment of the values of *ka* and *T* to obtain such a transition.

The results for the case of $\varepsilon _c=\text{ Re }\varepsilon _s$ are presented in Fig. 9c,d. Tunable spectral locations of $\text{ max }~\sigma _t$ for the modes of the types A and B (i.e., for both LSPRs and localized ENZ resonances) are possible, even though their effects in the cases shown in Fig. 9a,b tend to vanish. These modes can also contribute to various transition scenarios, provided that the effect of losses is compensated. The general feature connected with the increase of *T* is that the maxima of $\sigma _t$ become more blurred. Examples of various transitions are shown in Fig. 9c,d. One of transitions similar to 1a in Fig. 9a is denoted in Fig. 9c by 1b. Transitions from one mode of type A to the modes of type B are denoted in Fig. 9c,d by 2a, 2b, and 2c; the one from one of the modes of type A to another is denoted by 3 therein. Furthermore, there may be transitions from one mode of type B to another (not visible in Fig. 9), which need just very small changes of *T*. Interestingly, the slope of the lowest-*f* maximum and those of the modes of type A, on the one hand, and the slope of the modes of type B, on the other hand, can be close or not close, depending on *b*/*a*. Roughly speaking, the smaller *b*/*a* is, the larger the slope for type A will be. This observation is also confirmed by the results presented in Supplementary Information, Fig. S5 for the case of $b=13~\upmu \text{ m }$ and $a=14~\upmu \text{ m }$, as well as by the our other (not shown) results. At the same time, the slope is nearly constant for the modes of the type B.

**Figure 10:**
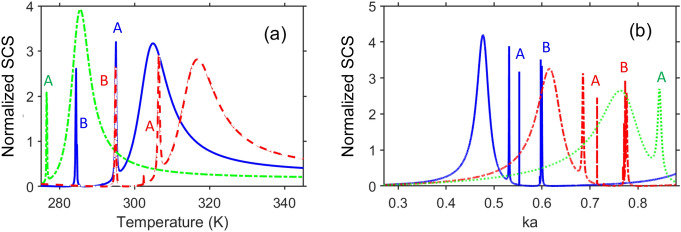
(**a**) $\sigma _t$ as a function of *T* for $\varepsilon _c=\text{ Re }\varepsilon _s$, when $ka=0.51$ (dash-dotted green line), $ka=0.633$ (solid blue line), and $ka=0.7155$ (dash-dotted red line); (**b**) $\sigma _t$ as a function of *ka* when $T=279.9$ K (solid blue line), $T=302.4$ K (dash-dotted red line), and $T=323.7$ K (dotted green line); geometrical parameters are the same as in Fig. 9a,c. A and B indicate the maxima yielded by high-Q modes of the corresponding types.

To further clarify which modal transitions may be possible, we first assume that $ka=\text{ const }$. The selected slices taken from Fig. 9c are presented in Fig. 10a. For example, for $ka=0.633$, the first sharp maximum of $\sigma _t$ is achieved at $T=284.4$ K (mode B). Then, the second maximum of $\sigma _t$ is observed when the temperature is increased up to $T=295$ K (mode A), while $\sigma _t\approx {0}$ between these two maxima. Finally, the wide maximum associated with the lowest-*f* LSPR is observed at $T=305$ K. Similar behavior is observed here for two other values of *ka*. Next, Fig. 10b presents the slices from Fig. 9c that are taken at $T=\text{ const }$. As mentioned above, the shift of all scattering peaks toward larger *ka* takes place while increasing *T*. It occurs because smaller *T* is needed to achieve a desired value of $\text{ Re }\varepsilon _s$ at smaller *ka*.

## Conclusion

To summarize, we investigated the weak and strong scattering of electromagnetic waves on thin-wall cylinders to find and explore the role of the localized ENZ resonators, in terms of both far- and near-field characteristics. As demonstrated numerically, both the localized ENZ resonances and LSPRs can yield strong scattering. They differ not only in the cylinder permittivity range where they appear but also in the specifics of the field distributions around and inside the cylinder. The localized ENZ resonances appear in the frequency range where $\text{ Re }\varepsilon$ is a bit smaller than zero. It has been shown that the suppression of high-Q superscattering modes connected with LSPRs and localized ENZ resonances plays a key role in the appearance of the wideband invisibility range. Just as one of the possible options, InSb has been considered as the cylinder material, while the gain impurities may be needed in this case to enable the strong-scattering regimes. Notably, transition from the metallic to the insulator material phase, which can be shown by InSb at a gradual temperature variation, necessarily leads to an ENZ regime at a particular frequency. As follows from the obtained results, cylinders with thin walls are needed for obtaining of a wide invisibility range and localized ENZ resonances, whereas the dispersion of the cylinder’s material permittivity may be crucial for obtaining diverse superscattering modes. Moreover, a higher contrast between the regimes of weak and strong scattering is obtained, because LSPRs and localized ENZ resonances may also appear at small such *ka* that the square of the scatterer’s cross section is still small enough to avoid significant scattering beyond the resonances. For the undoped material (in the considered case—InSb), the effects of the lowest-frequency LSPR are only seen in the scattering cross-section. This regime can appear at $2a\propto \lambda /10$, i.e., being deeply subwavelength. If the losses can be reduced toward lower or even near-zero levels by means of doping, regimes of invisibility and superscattering could also be observed at the frequencies, which are very close to each other, i.e., the sharp peaks of $\sigma _t$ that appear owing to high-Q resonances are embedded into the invisibility range. The connection of near- and far-field characteristics has been analyzed in the obtained results. Each spatial maximum of the field near/inside the cylinder corresponds to a far-field maximum at nearly the same angular position, so that in some senses the former can be said to be imaged. Assuming that sensitivity to the control parameter variations may remain nearly the same after doping, transitions between the strong scattering regimes yielded by LSPRs and those by the localized ENZ resonances, or vice versa, should be possible. The same is true regarding transitions between two different LSPRs or between two different localized ENZ resonances. The transitions between the regimes of strong scattering and invisibility are also possible for the undoped material that may need only a 15 K variation in temperature, being close to the natural environmental conditions. Various core’s material properties and effects of proximity are planned to be considered at the next steps of this research program. The emulation of the basic properties of superscattering modes connected with the localized ENZ resonances by using metallo-dielectric composites that comprise natural impurity-free low-loss materials is in progress.

## Methods

The applied approach is based on the Fourier-Bessel series. The incident wave is assumed to be *p*-polarized, and so the axial magnetic field is presented in the form of Fourier-Bessel series as follows:

2 $$\begin{aligned} H_z(r,\phi )= & {} \sum _{n=-\infty }^{\infty }(-i)^{n}[J_{n}(k_1r)+c_{n}H_{n}^{(2)}{(k_1r)}] e^{in\phi }, \hspace{1cm} r\ge {a}, \end{aligned}$$

3 $$\begin{aligned} H_z(r,\phi )= & {} \sum _{n=-\infty }^{\infty }(-i)^{n}b_{n}J_{n}(k_2r) e^{in\phi }, \hspace{1cm} r\le {b}, \end{aligned}$$

and

4 $$\begin{aligned} H_z(r,\phi )=\sum _{n=-\infty }^{\infty }(-i)^{n}[a_{n}J_{n}(k_3r)+d_{n}Y_{n}(k_3r)] e^{in\phi }, \hspace{1cm} a\ge {r}\ge {b}, \end{aligned}$$

where $J_{n}(x)$ and $Y_{n}(x)$ stand for Bessel and Neumann *n*th-order cylindrical functions, respectively; $H_{n}^{(2)}$ is a 2nd-kind Hankel function of the order *n*; $a_n$, $b_n$, $c_n$, and $d_n$ are unknown coefficients; $k_1=k_3=\omega /c$ and $k_2={\omega /c}\sqrt{{\varepsilon _2}{\mu _2}}$; $\phi$ is measured from the *x*-axis in the counter-clockwise direction; $i^2=-1$. Two remaining field components are given by $E_r=(-i/\omega \varepsilon \varepsilon _0{r})\partial H/\partial \phi$ and $E_{\phi }=(i/\omega \varepsilon \varepsilon _0)\partial H/\partial r$. Imposing the continuity condition for the tangential field components, $H_z$ and $E_{\phi }$, at $r=a$ and $r=b$, then taking into account the orthogonality of the exponential functions,

and finally rearranging the terms, we obtain

5 $$\begin{aligned}{} & {} b_{n}M_{n}(k_{2}b,k_3{b})=w_{3}d_{n}U_n(k_{3}b), \end{aligned}$$

6 $$\begin{aligned}{} & {} L_{n}(k_{1}a,k_{3}a)+c_{n}{\tilde{L}}_{n}(k_{3}a,k_{1}a)=w_{3}d_{n}T_{n}(k_{3}a), \end{aligned}$$

7 $$\begin{aligned}{} & {} b_{n}S_{n}(k_{2}b,k_{3}b)=w_{3}a_{n}X_n(k_{3}b), \end{aligned}$$

8 $$\begin{aligned}{} & {} F_{n}(k_{1}a,k_{3}a)+c_{n}G_{n}(k_1{a},k_{3}a)=w_{3}a_{n}W_{n},(k_{3}a), \end{aligned}$$

where $M_{n}(k_{2}b,k_3{b})=w_{2}J_{n}'(k_{2}b)J_{n}(k_{3}b)-w_{3}J_{n}(k_{2}b)J_{n}'(k_{3}b)$, $L_{n}(k_{1}a,k_{3}a)=w_{1}J_{n}'(k_{1}a)J_{n}(k_{3}a)-w_{3}J_{n}(k_{1}a)J_{n}'(k_{3}a)$, ${\tilde{L}}_{n}(k_{3}a,k_{1}a)=w_{1}J_{n}(k_{3}a)H_{n}^{(2)}{'(k_{1}a)}-w_{3}J_{n}'(k_{3}a)H_{n}^{(2)}(k_{1}a)$, $U_n(k_{3}b)=-X_n(k_{3}b)=2/(\pi {k_3}b)$, $S_{n}(k_{2}b,k_{3}b)=w_{2}J_{n}'(k_{2}b)Y_{n}(k_{3}b)-w_{3}J_{n}(k_{2}b)Y_{n}'(k_{3}b)$, $F_{n}(k_{1}a,k_{3}a)=w_{1}J_{n}'(k_{1}a)Y_{n}(k_{3}a)-w_{3}J_{n}(k_{1}a)Y_{n}'(k_{3}a)$, $G_{n}(k_1{a},k_{3}a)=w_{1}Y_{n}(k_{3}a)H_{n}^{(2)}{'(k_{1}a)}-w_{3}Y_{n}{'(k_{3}a)}H_{n}^{(2)}(k_{1}a)$, $T_{n}(k_{3}a)=-W_{n}(k_{3}a)=2/(\pi {k_3}a)$. It was assumed here that for the impedances normalized by the free-space impedance, we have $w_1=w_3=1$ and $w_2=\sqrt{\mu _2/\varepsilon _2}$. For this study, we take $\mu _2=1$ and $\varepsilon _2=\varepsilon _c$.

After some algebra, we obtain analytical expressions for the coefficients

9 $$\begin{aligned} c_{n}=\frac{L_{n}S_{n}-M_{n}F_{n}}{G_{n}M_{n}-{\tilde{L}}_{n}S_{n}}, \hspace{2cm} b_{n}=\frac{a}{b}\frac{L_{n}G_{n}-F_{n}\tilde{G_{n}}}{M_{n}G_{n}-S_{n}{\tilde{L}}_{n}}. \end{aligned}$$

Expressions for the coefficients $a_{n}$ and $d_{n}$ (not shown) have the same complexity as $b_n$ and $c_n$. Once all coefficients are found, the field components $H_{z}$, $E_{\phi }$, and $E_{r}$ can be calculated. To quantify scattering in far zone, the normalized total scattering cross section

10 $$\begin{aligned} \sigma _t=(ka)^{-1}\sum _{n=-\infty }^{\infty }c_{n}c_{n}^{*} \end{aligned}$$

is calculated, where the asterisk means a complex conjugate. It is normalized by that of the perfectly conducting cylinder of the radius *a*. Moreover, the knowledge of the coefficients $c_n$ is sufficient to calculate a $\phi$-dependent scattering cross section.

### Supplementary Information

Supplementary Information.

## Data Availability

All of the data generated or analyzed during this study are included in this article and its supplementary information files.

## References

[1] Schurig D, Mock JJ, Justice BJ, Cummer SA, Pendry JB, Starr AF, Smith DR (2006). Metamaterial electromagnetic cloak at microwave frequencies. Science.

[2] Kundtz N, Gaultney D, Smith DR (2010). Scattering cross-section of a transformation optics-based metamaterial cloak. New J. Phys..

[3] Zhu X, Feng L, Zhang P, Yin X, Zhang X (2013). One-way invisible cloak using parity-time symmetric transformation optics. Opt. Lett..

[4] Landy N, Smith DR (2013). A full-parameter unidirectional metamaterial cloak for microwaves. Nat. Mater..

[5] Chen P-Y, Soric J, Alù A (2012). Invisibility and cloaking based on scattering cancellation. Adv. Mater..

[6] Alù A, Engheta N (2008). Multifrequency optical invisibility cloak with layered plasmonic shells. Phys. Rev. Lett..

[7] Silveirinha M, Alù A, Engheta N (2008). Infrared and optical invisibility cloak with plasmonic implants based on scattering cancellation. Phys. Rev. B.

[8] Farhat M, Chen P-Y, Bagci H, Amra C, Guenneau S, Alu A (2015). Thermal invisibility based on scattering cancellation and mantle cloaking. Sci. Rep..

[9] Orazbayev B, Estakhri NM, Beruete M, Alù A (2015). Terahertz carpet cloak based on a ring resonator metasurface. Phys. Rev. B.

[10] Huang Y, Zhang J, Zhou J, Qiang B, Xu Z, Liu L, Tao J, Kossowski N, Wang Q, Luo Y (2021). Polarization-robust mid-infrared carpet cloak with minimized lateral shift. Photon. Res..

[11] Maegawa Y, Nakata Y, Sanada A (2023). All-dielectric carpet cloaks with three-dimensional anisotropy control. Nanophotonics.

[12] Nicorovici NAP, McPhedran RC, Enoch S, Tayeb G (2008). Finite wavelength cloaking by plasmonic resonance. New J. Phys..

[13] Alitalo P, Bongard F, Zürcher J-F, Mosig J, Tretyakov S (2009). Experimental verification of broadband cloaking using a volumetric cloak composed of periodically stacked cylindrical transmission-line networks. Appl. Phys. Lett..

[14] Rybin MV, Samusev KB, Kapitanova PV, Filonov DS, Belov PA, Kivshar YS, Limonov MF (2017). Switchable invisibility of dielectric resonators. Phys. Rev. B.

[15] Ruan Z, Fan S (2010). Superscattering of light from subwavelength nanostructures. Phys. Rev. Lett..

[16] Wang C, Qian C, Hu H, Shen L, Wang ZJ, Wang H, Xu Z, Zhang B, Chen H, Lin X (2020). Superscattering of light in refractive-index near-zero environments. Prog. Electromagn. Res..

[17] Huang Y, Gao L (2014). Superscattering of light from core-shell nonlocal plasmonic nanoparticles. J. Phys. Chem. C.

[18] Qian C, Lin X, Yang Y, Xiong X, Wang H, Li E, Kaminer I, Zhang B, Chen H (2019). Experimental observation of superscattering. Phys. Rev. Lett..

[19] Ruan Z, Fan S (2011). Design of subwavelength superscattering nanospheres. Appl. Phys. Lett..

[20] Liu W (2017). Superscattering pattern shaping for radially anisotropic nanowires. Phys. Rev. A.

[21] Zouros GP, Kolezas GD, Almpanis E, Tsakmakidis KL (2021). Three-dimensional giant invisibility to superscattering enhancement induced by Zeeman-split modes. ACS Photon..

[22] Alaee R, Safari A, Sandoghdar V, Boyd RW (2020). Kerker effect, superscattering, and scattering dark states in atomic antennas. Phys. Rev. Res..

[23] Kumar R, Kajikawa K (2020). Superscattering from cylindrical hyperbolic metamaterials in the visible region. Opt. Express.

[24] Liu YJ, Dong HY, Dong Z-G, Wang J (2022). Engineering multimode resonances for tunable multifrequency superscattering. Opt. Express.

[25] Valero AC, Shamkhi HK, Kupriianov AS, Tuz VR, Bobrovs V, Kivshar YS, Shalin AS (2022). Reaching the superscattering regime with BIC physics. J. Phys. Conf. Ser..

[26] Ye K-P, Pei W-J, Sa Z-H, Chen H, Wu R-X (2021). Invisible gateway by superscattering effect of metamaterials. Phys. Rev. Lett..

[27] Raad SH, Zapata-Rodríguez CJ, Atlasbaf Z (2019). Graphene-coated resonators with frequency-selective super-scattering and super-cloaking. J. Phys. D Appl. Phys..

[28] Garcia-Vidal FJ, Fernández-Domínguez AI, Martin-Moreno L, Zhang HC, Tang W, Peng R, Cui TJ (2022). Spoof surface plasmon photonics. Rev. Mod. Phys..

[29] Huidobro PA, Shen X, Cuerda J, Moreno E, Martin-Moreno L, Garcia-Vidal FJ (2014). Magnetic localized surface plasmons. Phys. Rev. X.

[30] Pors A, Moreno E, Martin-Moreno L, Pendry JB, Garcia-Vidal FA (2012). Localized spoof plasmons arise while texturing closed surfaces. Phys. Rev. Lett..

[31] She HY, Li LW, Martin OJ, Mosig JR (2008). Surface polaritons of small coated cylinders illuminated by normal incident TM and TE plane waves. Opt. Express.

[32] Wang Q, Rogers ETF, Gholipour B, Wang CM, Yuan G, Teng J, Zheludev NI (2016). Optically reconfigurable metasurfaces and photonic devices based on phase change materials. Nat. Photon..

[33] Serebryannikov AE, Alici KB, Ozbay E, Lakhtakia A (2018). Thermally sensitive scattering of terahertz waves by coated cylinders for tunable invisibility and masking. Opt. Express.

[34] Lewi T, Evans HA, Butakov NA, Schuller JA (2017). Ultrawide thermo-optic tuning of PbTe meta-atoms. Nano Lett..

[35] Lepeshov S, Krasnok A, Alù A (2019). Nonscattering-to-superscattering switch with phase-change materials. ACS Photon..

[36] Huang Y, Shen Y, Min C, Veronis G (2018). Switching photonic nanostructures between cloaking and superscattering regimes using phase-change materials. Opt. Mater. Express.

[37] Luo J, Li X, Zhang X, Guo J, Liu W, Lai Y, Zhan Y, Huang M (2021). Deep-learning-enabled inverse engineering of multi-wavelength invisibility-to-superscattering switching with phase-change materials. Opt. Express.

[38] Colak D, Nosich AI, Altintas A (1995). Radar cross-section study of cylindrical cavity-backed apertures with outer or inner material coating: the case of H-polarization. IEEE Trans. Antennas Propag..

[39] Serebryannikov AE, Nosich AI (2005). TE-case RCS analysis of finite-thickness slotted circular cylinder loaded with lossy filling. IEEE Trans. Antennas Propag..

[40] Kinsey N, DeVault C, Boltasseva A, Shalaev VM (2019). Near-zero index materials for photonics. Nat. Rev. Mater..

[41] Lobet M, Liberal I, Vertchenko L, Lavrinenko AV, Engheta N, Mazur E (2022). Momentum considerations inside near-zero index materials. Light Sci. Appl..

[42] Lobet M, Liberal I, Knall EN, Alam MZ, Reshef O, Boyd RW (2020). Fundamental radiative processes in near-zero-index media of various dimensionalities. ACS Photon..

[43] Torres V, Pacheco-Pena V, Rodríguez-Ulibarri P, Navarro-Cía M, Beruete M, Sorolla M, Engheta N (2013). Terahertz epsilon-near-zero graded-index lens. Opt. Express.

[44] Serebryannikov AE, Hajian H, Krawczyk M, Vandenbosch GAE, Ozbay E (2019). Embedded arrays of annular apertures with multiband near-zero-index behavior and demultiplexing capability at near-infrared. Opt. Mater. Express.

[45] Vassant S, Archambault A, Marquier F, Pardo F, Gennser U, Cavanna A, Pelouard J-L, Greffet J-J (2012). Epsilon-near-zero mode for active optoelectronic devices. Phys. Rev. Lett..

[46] Wu J, Xie ZT, Sha Y, Fu H, Li Q (2021). Epsilon-near-zero photonics: infinite potentials. Photon. Res..

[47] Serebryannikov AE, Skigin DC, Hajian H, Ozbay E (2023). Wide-angle and simultaneously wideband blazing (deflection) enabling multifunctionality in metagratings comprising epsilon-near-zero materials. J. Opt. Soc. Am. B.

[48] Chu H, Li Q, Liu B, Luo J, Sun S, Hang ZH, Zhou L, Lai Y (2018). A hybrid invisibility cloak based on integration of transparent metasurfaces and zero-index materials. Light Sci. Appl..

[49] Alu A, Silverinha MG, Salandrino A, Engheta N (2007). Epsilon-near-zero metamaterials and electromagnetic sources: Tailoring the radiation phase pattern. Phys. Rev. B.

[50] Wang C, Shi R, Gao L, Shalin AS, Luo J (2023). Quenching of second-harmonic generation by epsilon-near-zero media. Photon. Res..

[51] Kim J, Dutta A, Naik GV, Giles AJ, Bezares FJ, Ellis CT (2016). Role of epsilon-near-zero substrates in the optical response of plasmonic antennas. Optica.

[52] Pacheco, J., Jr. *Theory and Application of Left-Handed Metamaterials*, Ph.D. dissertation, Massachusetts Institute of Technology (2004).

[53] Valagiannopoulos CA (2007). Effect of cylindrical scatterer with arbitrary curvature on the features of a metamaterial slab antenna. Prog. Electromagn. Res..

[54] Valagiannopoulos CA (2011). High selectivity and controllability of a parallel-plate component with a filled rectangular ridge. Prog. Electromagn. Res..

[55] Khan I, Fang Z, Palei M, Lu J, Nordin L, Simmons EL, Dominguez O, Islam SM, Xing HG, Jena D (2020). Engineering the Berreman mode in mid-infrared polar materials. Opt. Express.

[56] Campione S, Brener I, Marquier F (2015). Theory of epsilon-near-zero modes in ultrathin films. Phys. Rev. B.

[57] Newman WD, Cortes CL, Atkinson J, Pramanik S, DeCorby RG, Jacob Z (2015). Ferrell–Berreman modes in plasmonic epsilon-near-zero media. ACS Photon..

[58] Wang J, Wang L, Liu J (2020). Overview of phase-change materials based photonic devices. IEEE Access.

[59] Wuttig M, Bhaskaran H, Taubner T (2017). Phase-change materials for non-volatile photonic applications. Nat. Photon..

[60] Jaffray W, Saha S, Shalaev VM, Boltasseva A, Ferrera F (2022). Transparent conducting oxides: From all-dielectric plasmonics to a new paradigm in integrated photonics. Adv. Opt. Photon..

[61] Park J, Kang J-H, Liu X, Brongersma ML (2015). Electrically tunable epsilon-near-zero (ENZ) metafilm absorbers. Sci. Rep..

[62] Foteinopoulou S, Devarapu GCR, Subramania GS, Krishna S, Wasserman D (2019). Phonon-polaritonics: Enabling powerful capabilities for infrared photonics. Nanophotonics.

[63] Serebryannikov AE, Nojima S, Alici KB, Ozbay E (2015). Effect of in-material losses on terahertz absorption, transmission, and reflection in photonic crystals made of polar dielectrics. J. Appl. Phys..

[64] Basharin AA, Mavidis C, Kafesaki M, Economou EN, Soukoulis CM (2013). Epsilon near zero based phenomena in metamaterials. Phys. Rev. B.

[65] Hajian H, Ghobadi A, Serebryannikov AE, Butun B, Vandenbosch GAE, Ozbay E (2019). Tunable infrared asymmetric light transmission and absorption via graphene-hBN metamaterials. J. Appl. Phys..

[66] Genç A, Patarroyo J, Sancho-Parramon J, Bastús NG, Puntes V, Arbiol J (2017). Hollow metal nanostructures for enhanced plasmonics: Synthesis, local plasmonic properties and applications. Nanophotonics.

[67] Iyer, P. P., Butakov, N. A. & Schuller, J. A. Reconfigurable semiconductor phased-array metasurfaces. *ACS Photon.* **2**, 1077–1084 (2015).

[68] Chiadini F, Fiumara V, Mackay TG, Scaglione A, Lakhtakia A (2017). Temperature-mediated transition from Dyakonov–Tamm surface waves to surface-plasmon-polariton waves. J. Opt..

[69] Serebryannikov AE, Lakhtakia A, Aalizadeh M, Ozbay E, Vandenbosch GAE (2018). Temperature-mediated invocation of the vacuum state for switchable ultrawide-angle and broadband deflection. Sci. Rep..

[70] Wan B, Zhang H, Wang P (2021). Nonreciprocal absorber with a narrow band of angular polarization sensitive regions based on a quasi-periodic structure. Opt. Lett..

[71] Oszwaldowski M, Zimpel M (1988). Temperature dependence of intrinsic carrier concentration and density of states effective mass of heavy holes in InSb. J. Phys. Chem. Solids.

[72] Liberal, I., Mahmoud, A. M. & Engheta, N. Geometry-invariant resonant cavities. *Nat. Commun.* **7**, 10989 (2016). 10.1038/ncomms10989 PMC4820806 27010103

[73] Kim Y, Lee S-Y, Ryu J-W, Kim I, Han J-H, Tae H-S, Choi M, Min B (2016). Designing whispering gallery modes via transformation optics. Nat. Photon..

[74] Yang Y-D, Huang Y-Z, Chen Q (2007). High-Q TM whispering-gallery modes in three-dimensional microcylinders. Phys. Rev. A.

[75] Zheng Y, Fang Z, Liu S, Cheng Y, Chen X (2019). High-Q exterior whispering-gallery modes in a double-layer crystalline microdisk resonator. Phys. Rev. Lett..

[76] Kaliteevski MA, Brand S, Abram RA, Kavokin A, Dang LS (2007). Whispering gallery polaritons in cylindrical cavities. Phys. Rev. B.

[77] Treyssède F, Gallezot M (2021). High-frequency leaky whispering-gallery modes in embedded elastic spheres. Phys. Rev. B.

[78] Yang JJ, Huang M, Yu J, Lin YZ (2011). Surface whispering gallery modes. Europhys. Lett..

