# Supplementary material for: Exploring localized ENZ resonances and their role in superscattering, wideband invisibility, and tunable scattering
Source: Sci Rep. 2024 Jan 18;14:1580. doi: 10.1038/s41598-024-51503-y (PMC10796346; doi:10.1038/s41598-024-51503-y)
Supplement: Supplementary file 1 — Supplementary Information. [file 41598_2024_51503_MOESM1_ESM.pdf]

Supplementary Information

Exploring localized ENZ resonances and their role in superscattering, wideband invisibility, and tunable scattering

A.E. Serebryannikov and E. Ozbay

In order to confirm that the features observed in Figs. 1, 2, 4 and 9 have regular, not accident nature, the numerical study has been carried out for multiple sets of *a* and *b*. The selected results are presented in Figs. S1-S5. Figure S1 shows σ_t vs. *ka* for the case of *a*=28μm and *a*=42μm; compare to Fig. 1. As seen, increase of *a* and, generally speaking, increase of the volume of the scatterer, lead to that the weak scattering occurs only in the vicinity of Re ε_c=1, whereas the signatures of the modes of type A and B (which are suppressed here by losses) are seen in the presented results. The effect of LSPRs (both the lowest-frequency mode and the modes referred to as the modes of the type A) in strong scattering is dominant at Re ε_c<0 in all cases, what leads to that the smaller *b*/*a* is, the larger σ_t can be achieved. Vice versa, at Re ε_c>1, the larger structure's volume is the dominant factor for obtaining a larger σ_t. It follows from the obtained results that smaller *a* and larger *b*/*a* yield a wide invisibility range, while strong scattering at Re ε_c<0 does remain.

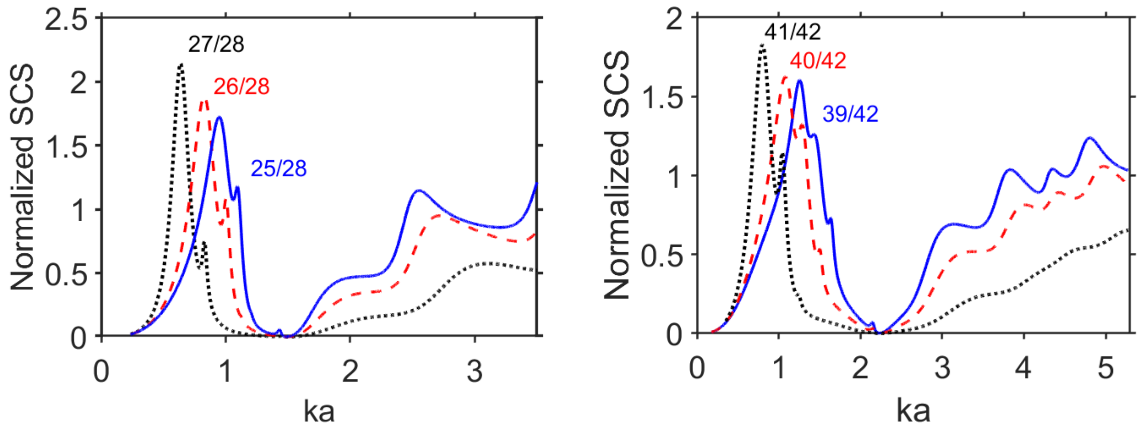

(a) (b)

Figure S1. Normalized total scattering cross section, σ_t, for thin-wall cylinders made of dispersive material with ε_c=ε_s for (a) *b*=27 μm (black dotted line), 26 μm (red dashed line), and 25 μm (blue solid line), *a*=28 μm; (b) *b*=41 μm (black dotted line), 40 μm (red dashed line), and 39 μm (blue solid line), *a*=42 μm; general geometry is the same as in the inset in Fig. 1(a). The ratios of *b*/*a* are shown near the curves.

Next, Fig. S2 presents σ_t as a function of *ka* when ε_c=Re ε_s and *a*=28μm. All features observed here are the same as those in Fig. 2. This is related to both lowest-frequency mode and modes of the types A and B. Similarly to the cases of *a*=14μm and *a*=42μm, we see here sharp peaks of σ_t that appear due to the modes of the types A and B, which are suppressed when the realistic losses are taken into account. The effect of losses on the appearance of these peaks is illustrated by Fig. S3; see Fig. 4 for the comparison. The results for the modes of the type B are shown in Fig. S3(a). It is obvious that |Im ε_c| should be three orders of magnitude smaller than |Im ε_s|, in order to warranty a significant effect of the two modes of the type B, which appear in the vicinity of *ka*=0.74 and *ka*=0.76. For the second of them, a 20-fold decrease of |Im ε_c| (*C*=0.05) is needed for the visibility of this mode in σ_t. At the same time, for the first of them, such an increase is yet insufficient; the details are shown in Fig. S3(b). To compare, Fig. S3(c) presents the results for the lowest-frequency maximum of σ_t. It is observed that

σ_t is weakly sensitive to the applied variations of *C*. The aforementioned features can be explained in terms of dark and bright modes, but this is beyond the scope. The situation is similar for the modes of the type A (not shown). For instance, for the maximum of σ_t at *ka*=0.633, *C*=0.5 (and even a larger *C*) can be sufficient to see the effect of this mode in σ_t, while *C*=0.002 warranties that it is seen almost to the same extent as for *C*=0. In turn, the mode arising at *ka*=0.6595 shows max σ_t >2.5 for *C*=0.002 and max σ_t >1 for *C*=0, whereas max σ_t <0.5 for *C*=0.005, 0.025, and 0.05.

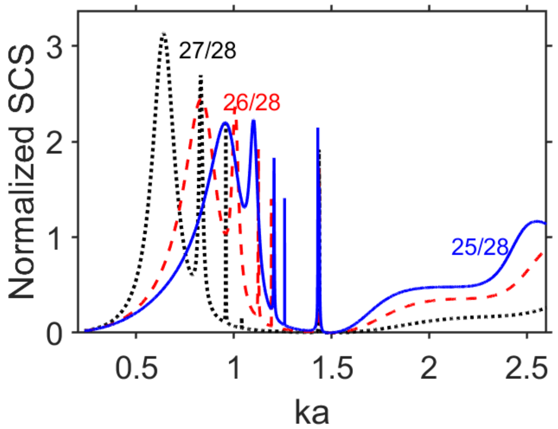

Figure S2. Normalized total scattering cross section, σ_t, for thin-wall cylinders made of dispersive material with ε_c=Re ε_s for *b*=27 μm (black dotted line), 26 μm (red dashed line), and 25 μm (blue solid line), *a*=28 mm; the ratios of *b*/*a* are shown near the curves.

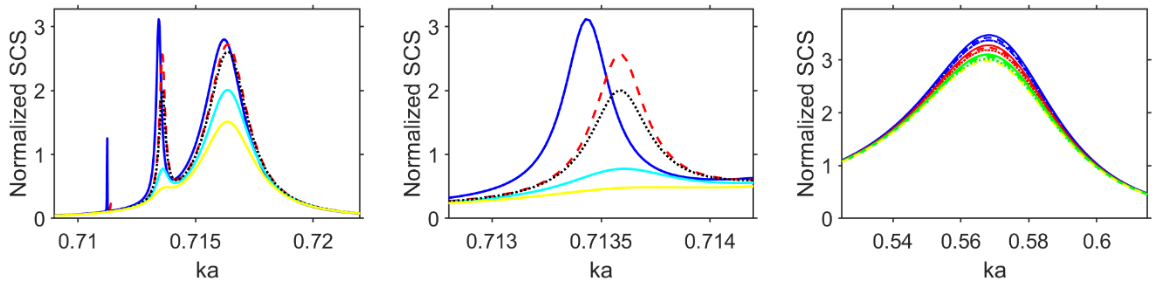

(a) (b) (c)

Figure S3. Normalized total scattering cross section, σ_t, at the selected values of *C* in Im ε_c=*C* Im ε_s: (a) *C*=0 (blue solid line), 0.002 (red dashed line), 0.005 (green dash-dotted line), 0.025 (black dotted line), 0.050 (violet dashed line), for the modes of type B; (b) fragment of (a) at the maximum of σ_t in the vicinity of *ka*=0.7135; (c) *C*=0.025, 0.05, 0.075 (blue curves), *C*=0.125, 0.15, 0.175 (red curves), *C*=0.225, 0.25, 0.275 (green curves), 0.3 (yellow curve), for the lowest-frequency resonance; *b*=11 μm and *a*=14 μm.

For the sake of completeness, we present the results for the gain case, i.e., Im ε_c is assumed to have the opposite sign than above. In Fig. S4, σ_t vs. *ka* is presented for the selected values of *C*, for one of the modes of the type B (vicinity of *ka*=0.716) and one of the modes of the type A (vicinity of *ka*=0.633). For the former, we observe the increase of σ_t up to 6.8 when *C*=-0.05. The possibility of a sharp increase of σ_t is expected to occur in the close vicinity of the complex eigenvalues; it needs an additional study. Notably, such a behavior has been observed for the other mode of the type B (in the vicinity of *ka*=0.713). In this case, max σ_t>14 is obtained *C*=-0.025 (not shown), assuming that *C* takes

the same values as in Fig. S4. In case of the lowest-frequency mode which has the lowest *Q*-factor among the studied modes, there is just a weak effect of *C* variable from 0 to -0.05, as in Fig. S4 (not shown). However, even for this mode larger max σ_t can be achieved. In particular, max σ_t>11 has been obtained in the simulations at *C*=-1.5. In line with the expectations of the effects of complex eigenvalues, we performed simulations in the vicinity of *ka*=0.633 (mode of the type A) and *ka*= 0.716 (mode of type B). The results obtained for the former show that max σ_t>29 when *C*=-0.1. At the same time, much smaller values of max σ_t are obtained when *C*=-1.0, -0.7, -0.5, and -0.2, so the optimum *C* does exist. For the latter, max σ_t>35 when *C*=-0.1, and then it becomes smaller when σ_t takes the same values as above, i.e., from -1.0 to -0.2.

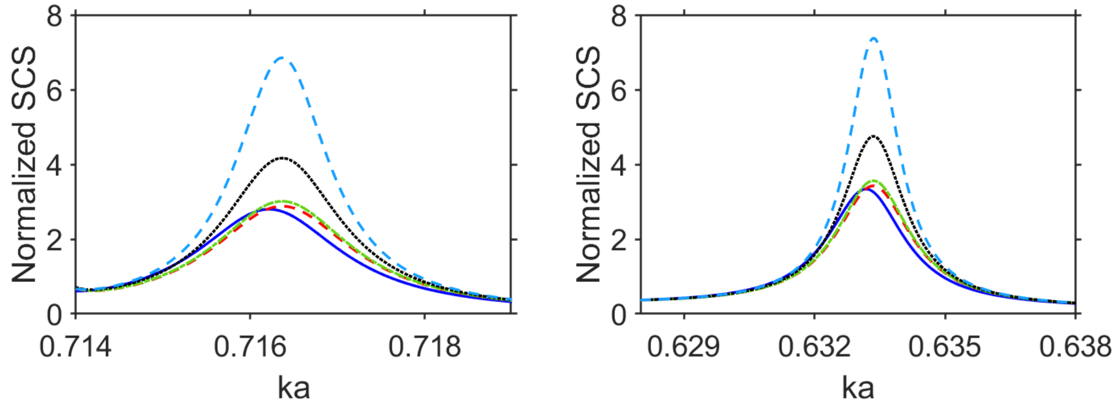

(a) (b)

Figure S4. Normalized total scattering cross section, σ_t, at selected values of *C* in Im ε_c=*C* Im ε_s, (a) *C*=0 (blue solid line), -0.002 (red dashed line), -0.005 (green dash-dotted line), -0.025 (black dotted line), and -0.05 (light-blue dashed line), for one of the modes of type B; (b) same but for one of the modes of type A; *b*=11 μm and *a*=14 μm.

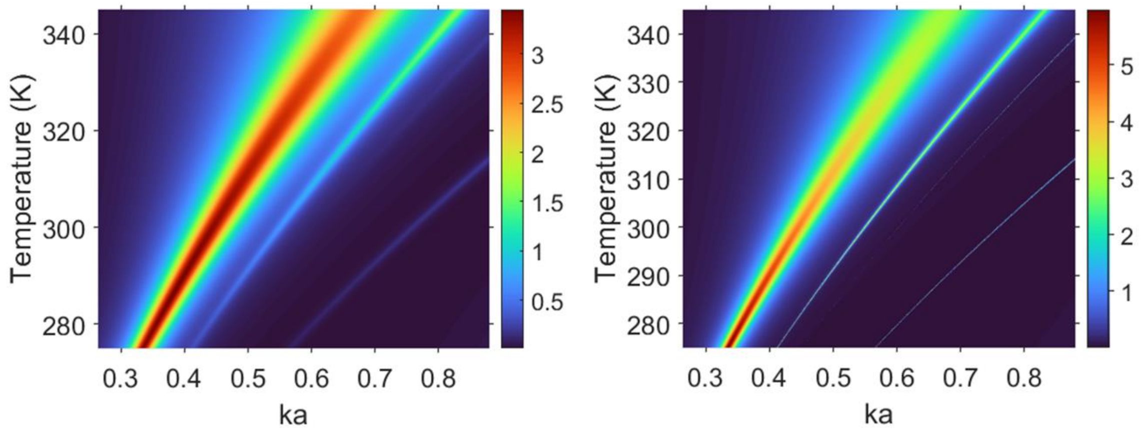

(a) (b)

Figure S5. Normalized total scattering cross section, σ_t, in (*ka*,*T*)-plane for *b*=13 μm and *a*=14 μm, (a) ε_c=ε_s, (b) ε_c=Re ε_s .

Finally, in Fig. S5, the numerical results are presented for σ_t in (*ka*,*T*)-plane for a thinner-wall cylinder than in Fig. 9. The results indicate that no new feature appears when *b*=11 mm is changed for *b*=13 mm. The only significant difference is the slope of the “mountain” of max σ_t. Indeed, the slope in Fig. S5 is larger than that in Fig. 9(a) but similar to that in Fig. 9(b). This means that the same increments ΔT lead to either smaller or larger changes in spectral location of max σ_t, depending on *b*/*a*

at a=const, for the lowest-frequency mode and the modes of the type A. An additional study is needed in this case, in order to quantify the slope as a function of b/a and a. On the other hand, for the modes of the type B, the slope is determined rather by the material properties. Generally speaking, the spectral distance between the ranges occupied by the modes of the types A and B can depend on b/a and T.
